# Association between phenotypic age and mortality risk in individuals with obesity: a retrospective cohort study

**DOI:** 10.3389/fpubh.2024.1505066

**Published:** 2024-12-09

**Authors:** Yingxuan Huang, Apei Zhou, Yisen Huang, Yubin Wang, Xiaobo Liu, Xiaoqiang Liu

**Affiliations:** ^1^Department of Gastroenterology, First Hospital of Quanzhou Affiliated to Fujian Medical University, Quanzhou, Fujian, China; ^2^McConnell Brain Imaging Centre, Montreal Neurological Institute, McGill University, Montreal, QC, Canada

**Keywords:** phenotypic age acceleration, obesity, all-cause mortality, cardiovascular disease, cancer, NHANES

## Abstract

**Objective:**

This study investigates the association between phenotypic age acceleration (PAA) and all-cause and cause-specific mortality in obese individuals.

**Methods:**

Data were drawn from the National Health and Nutrition Examination Survey (NHANES) between 1999 and 2018, including 9,925 obese adults (BMI ≥ 30 kg/m^2^). PAA, defined as phenotypic age exceeding chronological age, was assessed using clinical biomarkers. Kaplan-Meier survival analysis and Cox proportional hazards models were used to assess the relationship between PAA and all-cause, cardiovascular, and cancer mortality, adjusting for covariates such as age, gender, race, lifestyle, and health status. Subgroup and sensitivity analyses were performed to ensure the robustness of the findings.

**Results:**

During a median follow-up of 10.6 years, 1,537 deaths were recorded, including 419 from cardiovascular disease and 357 from cancer. PAA was significantly associated with all-cause mortality (HR = 1.84, 95% CI: 1.64–2.06), cardiovascular mortality (HR = 1.86, 95% CI: 1.50–2.31), and cancer mortality (HR = 1.47, 95% CI: 1.17–1.85). These associations remained significant after adjusting for multiple variables, and sensitivity analyses confirmed the robustness of the results.

**Conclusion:**

PAA is an independent predictor of all-cause, cardiovascular, and cancer mortality in obese individuals. This study highlights the importance of PAA in mortality risk assessment and health management in the obese population.

## 1 Introduction

Obesity is a complex metabolic disease and has become a significant global public health issue, placing a heavy burden on healthcare systems (1, 2). Obesity is closely associated with the development of various chronic diseases, such as diabetes, cardiovascular diseases, and cancer, and it is also a major cause of premature death (3, 4). Despite considerable advancements in understanding the pathophysiology of obesity and its treatment strategies, mortality rates among obese individuals remain high (5). Therefore, identifying biomarkers that are closely associated with mortality risk in obese individuals is critical for timely identification of high-risk groups and the implementation of effective interventions.

Biological aging, a process characterized by a gradual decline in the body's homeostatic capabilities, is considered a key factor in the worsened prognosis associated with obesity (6, 7). However, the rate of aging varies significantly among individuals, and traditional risk assessments based solely on chronological age (CA) often fail to accurately capture an individual's true physiological condition (8). Phenotypic age acceleration (PAA) refers to the extent of biological aging beyond CA, with a positive value indicating that an individual's biological age (BA) exceeds their actual age (9). Previous studies have demonstrated that PAA is strongly associated with all-cause and cardiovascular-related mortality in patients with various chronic diseases, such as coronary artery disease and diabetes (10, 11). However, it remains unclear whether PAA is associated with mortality risk in obese individuals.

This study aims to explore the association between PAA and both all-cause mortality as well as cause-specific mortality, such as cardiovascular and cancer-related deaths, in obese individuals. By analyzing these BA markers, we hope to provide clinicians with more effective tools to help identify high-risk individuals within the obese population and optimize the implementation of related interventions.

## 2 Methods

### 2.1 Study population

**Figure 1:**
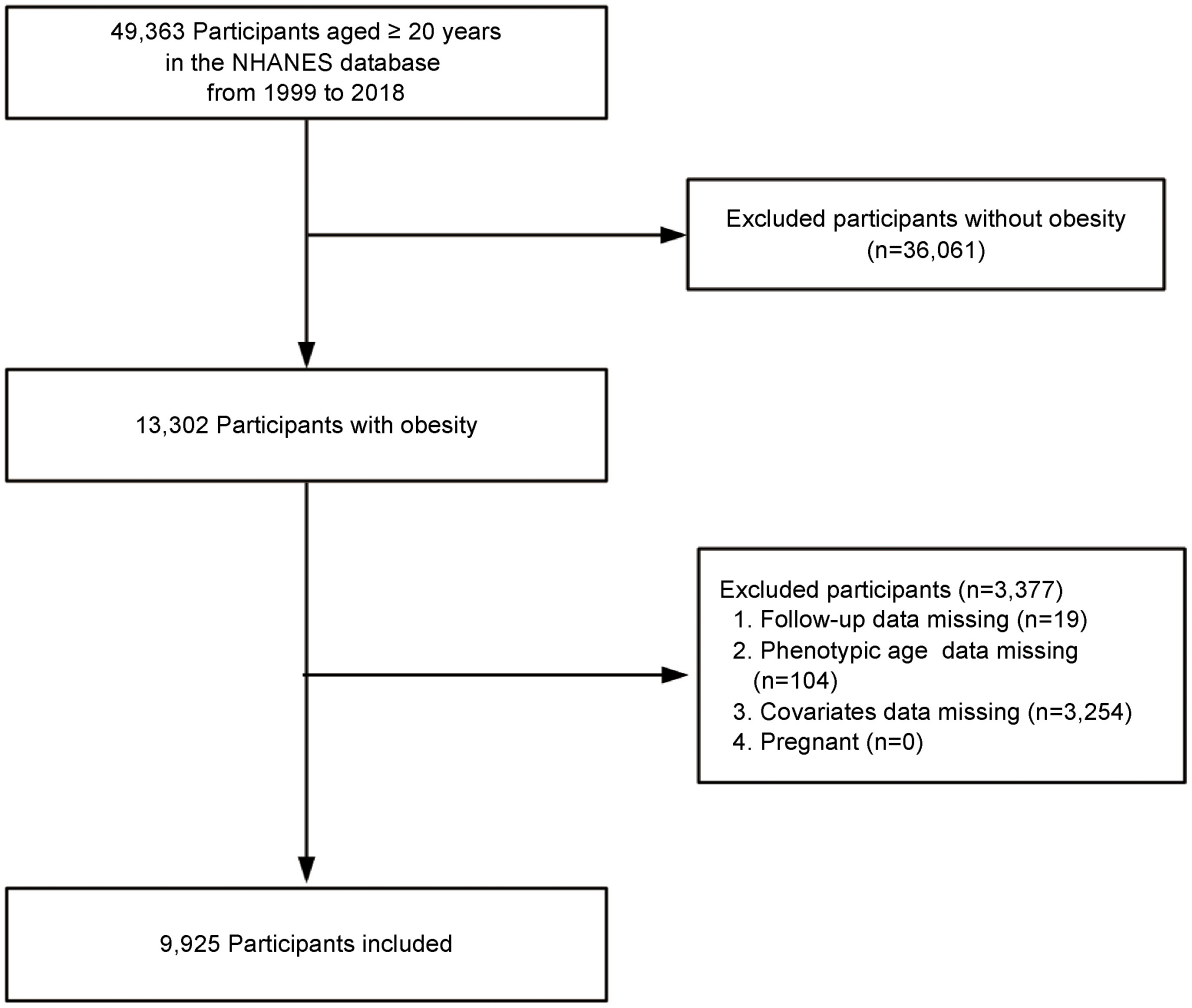
Study flow chart.

This study utilized data from obese adult participants in the National Health and Nutrition Examination Survey (NHANES) conducted between 1999 and 2018. NHANES is a nationally representative survey conducted by the National Center for Health Statistics (NCHS), using a stratified, multistage probability sampling method to assess the health and nutritional status of the non-institutionalized U.S. population. Adults aged ≥20 years with obesity, defined as a body mass index (BMI) ≥30 kg/m^2^, were included in the study. Exclusions were made for participants who were lost to follow-up, those with missing laboratory data required to calculate phenotypic age (PA), those with missing covariate data, and pregnant women. Ultimately, the study sample included 9,925 obese adults (Figure 1). The NHANES study protocol was approved by the NCHS Research Ethics Review Board, and all participants provided written informed consent at enrollment. This study followed the Strengthening the Reporting of Observational Studies in Epidemiology (STROBE) guidelines for reporting observational studies. The Ethics Committee of First Hospital of Quanzhou Affiliated to Fujian Medical University approved the study (decision no. 2024-184).

### 2.2 Measurement of PAA

PA was estimated using multiple physiological markers obtained from laboratory analysis of blood samples, including 10 aging-related variables: chronological age (CA), albumin, creatinine, glucose, C-Reactive Protein (CRP), lymphocyte percentage, mean cell volume, red cell distribution width, alkaline phosphatase, and white blood cell count (12). The detailed method for calculating PA is provided in Supplementary material. The residual of PAA was calculated through linear regression of PA on CA. PAA reflects the difference between an individual's BA and CA, with those having a positive residual classified as experiencing “PAA,” and those with a negative residual classified as having “without PAA” (13).

### 2.3 Mortality determination

The survival status of participants was determined through the NHANES publicly available linked mortality files, with a cutoff date of December 31, 2019. Causes of death were classified based on the International Classification of Diseases, 10th Revision (ICD-10), focusing on all-cause mortality, cardiovascular disease mortality (ICD-10: I00-I09, I11, I13, I20-I51), and cancer mortality (ICD-10: C00-C97).

### 2.4 Covariate assessment

Based on previous studies, the potential covariates included age, gender, race, marital status, poverty income ratio (PIR), educational level, Healthy Eating Index (HEI-2015), physical activity, smoking status, alcohol intake, cardiovascular disease (CVD), hypertension, hyperlipidemia, diabetes, and cancer diagnosis (9, 14, 15). Specific definitions were as follows: age was treated as a continuous variable, recording the participants' actual age. Gender was self-reported; race was categorized as non-Hispanic White, others (non-Hispanic Black, Mexican American, other Hispanic, and other races). Marital status was categorized as married/cohabitating or unmarried/other (including widowed, divorced, or separated); PIR was divided into 1–1.3, 1.31–3.50, and >3.50 (16). Educational levels were divided into less than high school, high school or equivalent, and more than high school; HEI-2015 score evaluated diet quality based on 13 components, summing to a 100-point score indicating adherence to 2015–2020 Dietary Guidelines (17). Physical activity time was a continuous variable indicating time spent on activities such as walking, cycling, work, and recreational activities in a week, and PA was categorized into three levels: inactive (0 MET-min/week), insufficiently active (1-599 MET-min/week), and sufficiently active (≥600 MET-min/week) (18).Smoking status was categorized as never smokers (fewer than 100 cigarettes in their lifetime), former smokers (over 100 cigarettes but not currently smoking), and current smokers (over 100 cigarettes and currently smoking sometimes or daily) (19). Participants were categorized by alcohol intake into never drinkers (< 12 drinks in a lifetime), former drinkers (≥12 drinks in a year but not in the past year), and current drinkers (≥12 drinks in any given year and drank in the past year) (20). CVD is defined as a self-reported diagnosis of coronary heart disease, angina, stroke, myocardial infarction, or congestive heart failure (21). Hypertension was defined as a mean systolic blood pressure ≥140 mmHg and/or diastolic blood pressure ≥90 mmHg, self-reported diagnosis, or use of antihypertensive medications (19). Hyperlipidemia was defined by any of the following: (1) use of lipid-lowering drugs; (2) triglycerides ≥150 mg/dl; or (3) high cholesterol (total cholesterol ≥200 mg/dl, or LDL ≥130 mg/dl, or HDL < 40 mg/dl) (22). Diabetes was defined by a doctor's diagnosis, HbA1c ≥6.5%, fasting glucose ≥7.0 mmol/L, random glucose/Oral Glucose Tolerance Test (OGTT) ≥11.1 mmol/L, or use of diabetes medication/insulin (23). Cancer diagnosis was based on an affirmative response to the question, “Have you ever been told by a doctor or other health professional that you had cancer or any type of malignancy?” (18).

### 2.5 Statistical analysis

All analyses were conducted using R statistical software (version 4.2.1; http://www.R-project.org, The R Foundation) and Free Statistics software (version 1.7; Beijing, China, http://www.clinicalscientists. cn/freestatistics). Categorical variables were expressed as percentages, and continuous variables as means (standard deviation). Group differences were compared using the Chi-square test and two-sample independent *t*-test. Survival analyses were conducted using Kaplan-Meier survival curves to estimate all-cause, CVD, and cancer mortality in different PAA groups, with group differences assessed by the Log-rank test. To further analyze the relationship between PAA and mortality, Cox proportional hazards regression models were used to calculate hazard ratios (HRs) and their 95% confidence intervals (CIs). The covariates included in the model were determined based on clinical expertise, univariate screening results (*P*-value < 0.05), or whether they led to a change in the effect estimate >10%. The multivariable models were progressively adjusted for covariates: Model 1 adjusted for age and gender; Model 2 further adjusted for race, marital status, PIR, educational level, HEI-2015, physical activity, smoking status, and alcohol intake; Model 3 further adjusted for CVD, hypertension, hyperlipidemia, diabetes, and cancer. Restricted cubic spline (RCS) models were used to estimate and visualize the dose-response relationship between age acceleration residual and risks of all-cause and cause-specific mortality.

To assess the consistency of the relationship between PAA and all-cause mortality across different populations, subgroup analyses were performed for age, gender, smoking, alcohol intake, CVD, hypertension, hyperlipidemia, and diabetes. Sensitivity analyses were conducted to validate the robustness of the results: (1) excluding participants who died within 2 years of follow-up; (2) excluding participants with a baseline history of CVD; and (3) excluding participants with a baseline history of cancer. Cox regression models were re-fitted for each sensitivity analysis to evaluate the relationship between PAA and all-cause mortality; (4) Following NHANES analytic guidelines, we incorporated the complex sampling design and mobile examination center (MEC) exam sample weights into our study. Detailed information regarding the weighted analysis can be found in the Supplementary material. Specifically, in the Cox regression analysis, we used a weighted data model to conduct an in-depth analysis of the relationship between PAA and all-cause mortality, as well as cause-specific mortality, in the obese population.

## 3 Results

### 3.1 Participant characteristics

In Table 1, among the 9,925 obese participants, 5,496 (55.4%) were classified as the without PAA group, while 4,429 (44.6%) were in the PAA group. The age acceleration residual for the without PAA group was −5.5 ± 3.5, compared to 8.4 ± 9.3 in the PAA group (*P* < 0.001). In terms of covariates, the PAA group had a higher mean age (52.1 vs. 50.0 years, *P* < 0.001). The proportion of non-Hispanic Whites was higher in the without PAA group (48.9 vs. 41.9%, *P* < 0.001), and a greater percentage of individuals in the without PAA group were married or cohabiting with a partner (65.2 vs. 58.3%, *P* < 0.001). Additionally, the PAA group had a higher proportion of low-income individuals and current smokers, while the without PAA group had a higher Healthy Eating Index score (*P* < 0.001).

**Table 1:** Baseline characteristics of individuals with obesity in NHANES 1999–2018.

**Variables**	**Total**	**Without PAA**	**PAA**	***P*-value**
**Number of participants**	9,925	5,496	4,429	
**Age, mean** **±SD, year**	50.9 ± 16.4	50.0 ± 16.2	52.1 ± 16.6	< 0.001
**Gender**, ***n*** **(%)**				0.144
Male	4,540 (45.7)	2,478 (45.1)	2,062 (46.6)	
Female	5,385 (54.3)	3,018 (54.9)	2,367 (53.4)	
**Race**, ***n*** **(%)**				< 0.001
Non-Hispanic White	4,540 (45.7)	2,685 (48.9)	1,855 (41.9)	
Others	5,385 (54.3)	2,811 (51.1)	2,574 (58.1)	
**Marital status**, ***n*** **(%)**				< 0.001
Married/living with partner	6,169 (62.2)	3,585 (65.2)	2,584 (58.3)	
Never married/other	3,756 (37.8)	1,911 (34.8)	1,845 (41.7)	
**PIR group**, ***n*** **(%)**				< 0.001
1–1.3	2,924 (29.5)	1,473 (26.8)	1,451 (32.8)	
1.31–3.50	3,999 (40.3)	2,169 (39.5)	1,830 (41.3)	
>3.50	3,002 (30.2)	1,854 (33.7)	1,148 (25.9)	
**Education level**, ***n*** **(%)**				0.075
Less than high school	2,602 (26.2)	1,489 (27.1)	1,113 (25.1)	
High school or equivalent	2,478 (25.0)	1,345 (24.5)	1,133 (25.6)	
Above high school	4,845 (48.8)	2,662 (48.4)	2,183 (49.3)	
**HEI-2015, mean** **±SD**	51.5 ± 12.8	52.0 ± 12.8	50.9 ± 12.9	< 0.001
**Smoking status**, ***n*** **(%)**				< 0.001
Never	5,292 (53.3)	3,056 (55.6)	2,236 (50.5)	
Former	2,840 (28.6)	1,563 (28.4)	1,277 (28.8)	
Current	1,793 (18.1)	877 (16.0)	916 (20.7)	
**Alcohol intake**, ***n*** **(%)**				0.001
Never	1,335 (13.5)	772 (14.0)	563 (12.7)	
Former	2,320 (23.4)	1,213 (22.1)	1,107 (25.0)	
Current	6,270 (63.2)	3,511 (63.9)	2,759 (62.3)	
**Physical activity**, ***n*** **(%)**				< 0.001
Inactive	2,963 (29.9)	1,503 (27.3)	1,460 (33)	
Insufficiently active	2,581 (26.0)	1,688 (30.7)	893 (20.2)	
Sufficiently active	4,381 (44.1)	2,305 (41.9)	2,076 (46.9)	
**CVD**, ***n*** **(%)**				< 0.001
No	8,537 (86.0)	4,972 (90.5)	3,565 (80.5)	
Yes	1,388 (14.0)	524 (9.5)	864 (19.5)	
**Hypertension**, ***n*** **(%)**				< 0.001
No	4,489 (45.2)	2,772 (50.4)	1,717 (38.8)	
Yes	5,436 (54.8)	2,724 (49.6)	2,712 (61.2)	
**Hyperlipidemia**, ***n*** **(%)**				0.686
No	1,809 (18.2)	994 (18.1)	815 (18.4)	
Yes	8,116 (81.8)	4,502 (81.9)	3,614 (81.6)	
**Diabetes**, ***n*** **(%)**				< 0.001
No	7,331 (73.9)	4,719 (85.9)	2,612 (59.0)	
Yes	2,594 (26.1)	777 (14.1)	1,817 (41.0)	
**Cancer**, ***n*** **(%)**				< 0.001
No	8,990 (90.6)	5,052 (91.9)	3,938 (88.9)	
Yes	935 (9.4)	444 (8.1)	491 (11.1)	
**Age acceleration residual, mean** **±SD, year**	0.7 ± 9.7	−5.5 ± 3.5	8.4 ± 9.3	< 0.001

PAA, phenotypic age acceleration; HEI-2015, Healthy Eating Index-2015; PIR, poverty income ratio; CVD, cardiovascular disease.

### 3.2 Relationship between PAA and mortality

**Figure 2:**
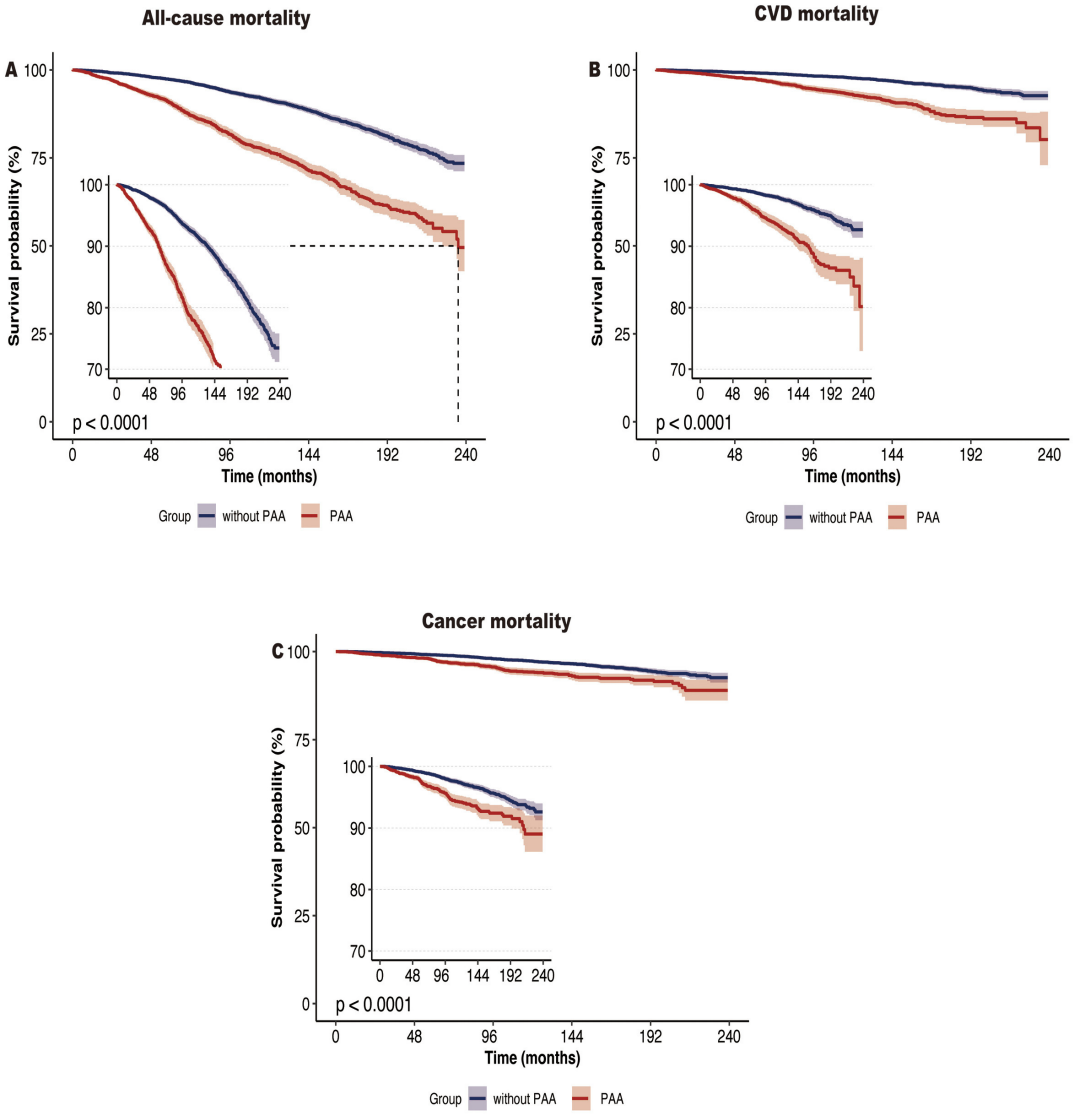
Kaplan-Meier survival curve of all-cause mortality **(A)**, CVD mortality **(B)**, and cancer mortality **(C)** according to phenotypic age acceleration among obese adults. CVD, cardiovascular disease.

**Figure 3:**
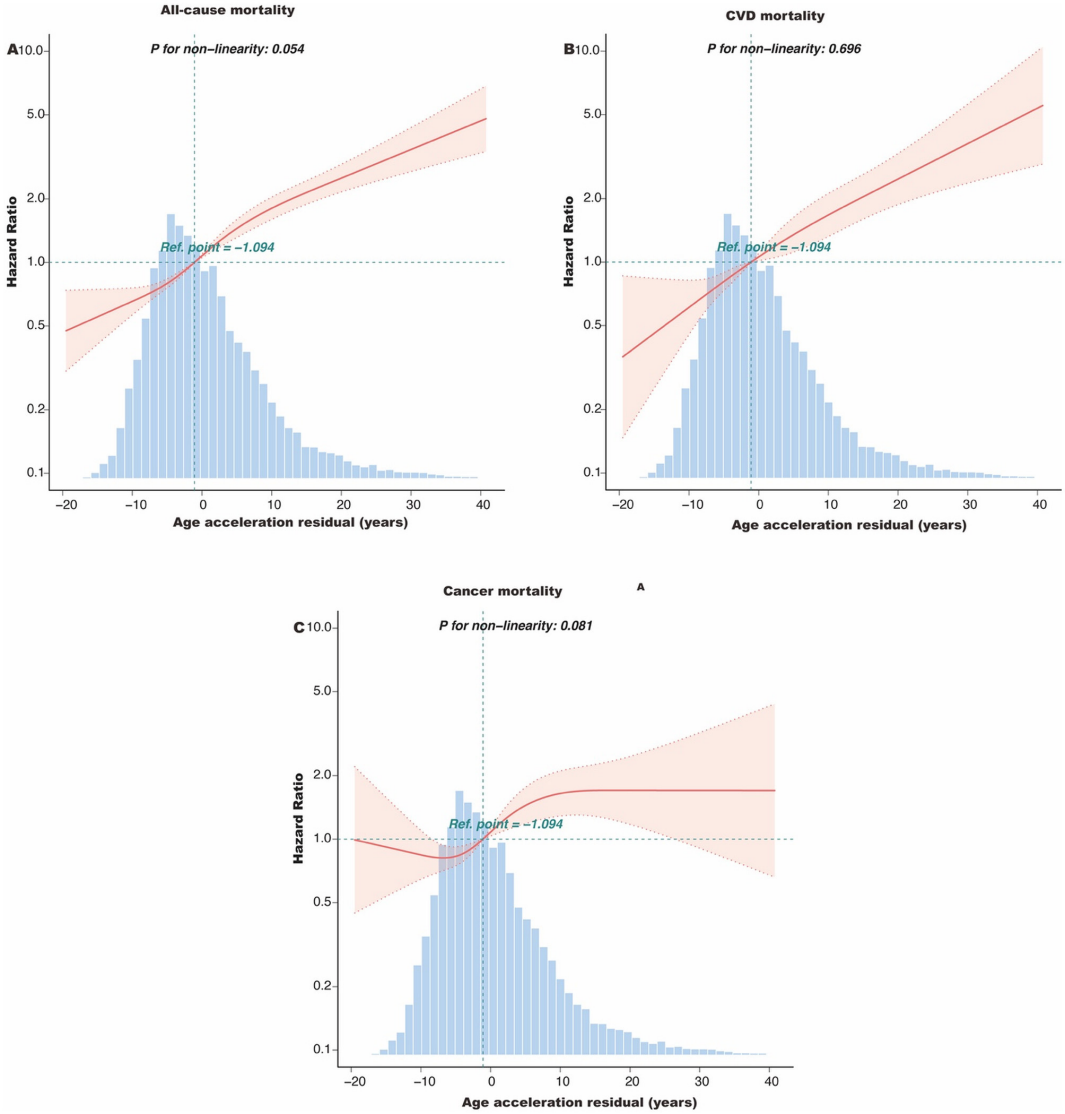
The dose-response association of the age acceleration residual with all-cause mortality **(A)**, CVD mortality **(B)**, and cancer mortality **(C)** among obese adults. This spline model was adjusted for age, gender, race, marital status, PIR group, educational level, HEI-2015, physical active, smoking status, and alcohol intake, CVD, hypertension, hyperlipidemia, diabetes, and cancer. HEI-2015, Healthy Eating Index-2015; PIR, poverty income ratio; CVD, cardiovascular disease.

During the median follow-up of 10.6 years (interquartile range: 4–14.7 years), a total of 1,537 participants died, including 419 deaths from CVD and 357 deaths from cancer. Kaplan-Meier survival analysis showed that participants with PAA had significantly higher all-cause mortality, CVD mortality, and cancer mortality compared to those without PAA (Figure 2, all *P*-values < 0.001). Table 2 shows that, after adjusting for age, gender, race, marital status, PIR, educational level, physical activity, smoking status, alcohol intake, CVD, hypertension, diabetes, and cancer, PAA remained significantly associated with all-cause mortality, CVD mortality, and cancer mortality. Compared to participants without PAA, those with PAA had an 84% increased risk of all-cause mortality (Model 3, HR = 1.84, 95%CI: 1.64–2.06, *P* < 0.001), an 86% increased risk of CVD mortality (Model 3, HR = 1.86, 95%CI: 1.50–2.31, *P* < 0.001), and a 47% increased risk of cancer mortality (Model 3, HR = 1.47, 95%CI: 1.17–1.85, *P* < 0.001). Age acceleration residual (measured in years) significantly increased the risk of all-cause mortality (HR = 1.04 per 1-year increase), CVD mortality (HR = 1.04–1.05 per 1-year increase), and cancer mortality (HR = 1.02–1.03 per 1-year increase), with all models reaching statistical significance (*P* < 0.001). As shown in Figure 3, the restricted cubic spline regression model indicated a linear positive correlation between PAA and all-cause mortality, CVD mortality, and cancer mortality (all *P*-values > 0.05).

**Table 2:** Adjusted hazard ratios of phenotypic age acceleration with risk of all-cause mortality and cause-specific mortality.

**Characteristics**	**Age acceleration residual**	***P*-value**	**Phenotypic age acceleration**		***P*-value**
			**No**	**Yes**	
**All-cause mortality**					
No. deaths/total (%)	1,537/9,925 (15.5)		793/5,496 (14.4)	744/4,429 (16.8)	
Model 1	1.04 (1.03–1.04)	< 0.001	1 (reference)	2.38 (2.15–2.64)	< 0.001
Model 2	1.04 (1.03–1.04)	< 0.001	1 (reference)	2.13 (1.92–2.36)	< 0.001
Model 3	1.04 (1.03–1.04)	< 0.001	1 (reference)	1.84 (1.64–2.06)	< 0.001
**CVD mortality**					
No. deaths/total (%)	419/9,925 (4.2)		206/5,496 (3.7)	213/4,429 (4.8)	
Model 1	1.05 (1.04–1.05)	< 0.001	1 (reference)	2.55 (2.09–3.11)	< 0.001
Model 2	1.05 (1.04–1.05)	< 0.001	1 (reference)	2.27 (1.86–2.78)	< 0.001
Model 3	1.04 (1.03–1.05)	< 0.001	1 (reference)	1.86 (1.50–2.31)	< 0.001
**Cancer mortality**					
No. deaths/total (%)	357/9,925 (3.8)		218/5,496 (4.0)	157/4,429 (3.5)	
Model 1	1.03 (1.02–1.04)	< 0.001	1 (reference)	1.74 (1.41–2.15)	< 0.001
Model 2	1.03 (1.02–1.04)	< 0.001	1 (reference)	1.58 (1.28–1.96)	< 0.001
Model 3	1.02 (1.01–1.03)	< 0.001	1 (reference)	1.47 (1.17–1.85)	< 0.001

Model 1 was adjusted for age and gender. Model 2 was additionally adjusted for race, marital status, PIR group, educational level, HEI-2015, physical active, smoking status, and alcohol intake. Model 3 was additionally adjusted for CVD, hypertension, hyperlipidemia, diabetes, and cancer.

HEI-2015, Healthy Eating Index-2015; PIR, poverty income ratio; CVD, cardiovascular disease.

### 3.3 Subgroup analysis

**Figure 4:**
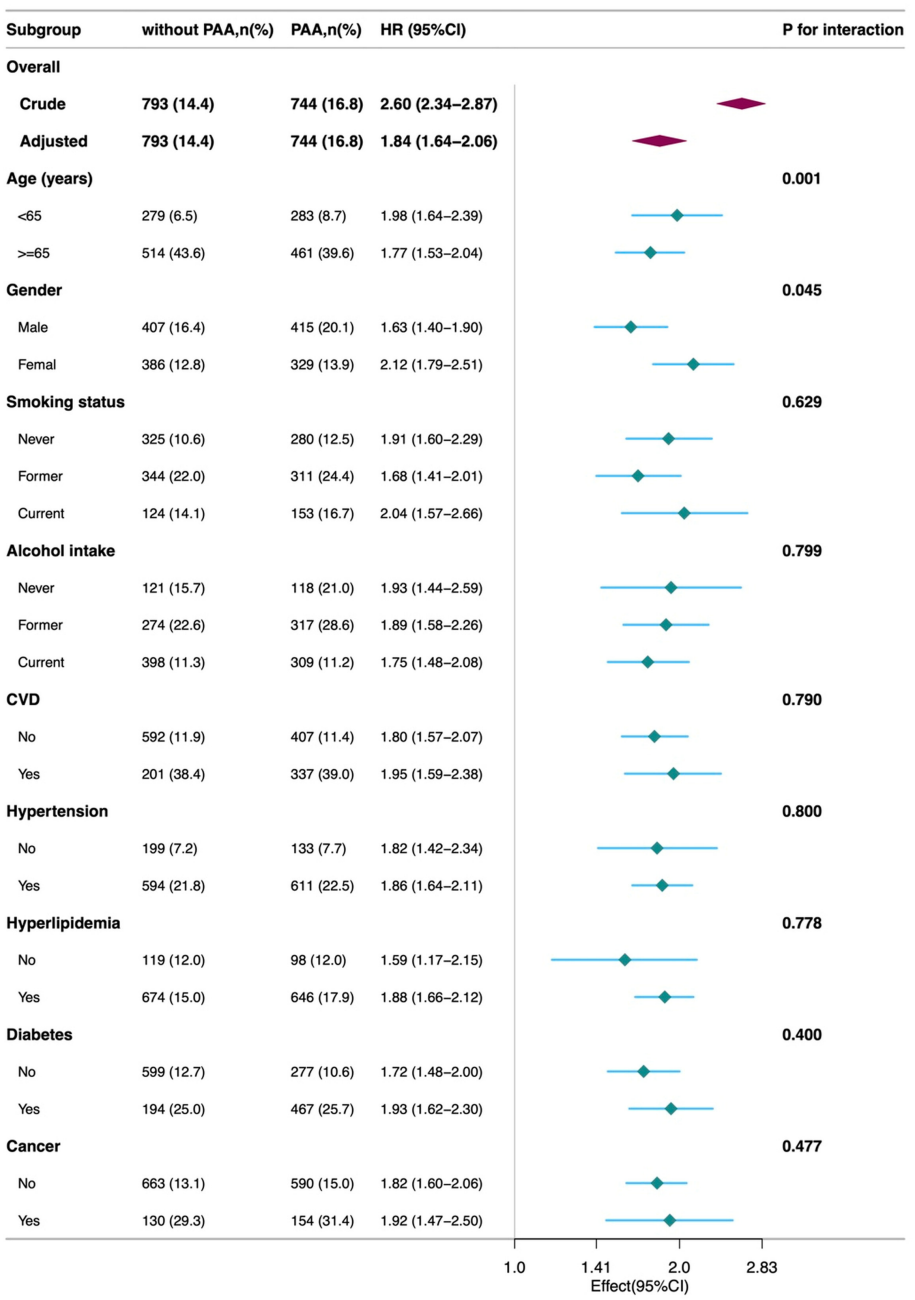
Subgroup analyses of the association of the phenotypic age acceleration with all-cause mortality among obese adults. CVD, cardiovascular disease; PAA, phenotypic age acceleration.

Figure 4 shows that the association between PAA and all-cause mortality was consistent across different subgroups based on age, gender, smoking, alcohol intake, CVD, hypertension, hyperlipidemia, and diabetes. Specifically, the all-cause mortality rate was significantly higher in the PAA group across all subgroups. A significant interaction was observed in the age subgroup and gender subgroup.

### 3.4 Sensitivity analysis

Several sensitivity analyses were conducted to validate the robustness of the results. First, after excluding participants who died within the first 2 years of follow-up, the association between PAA and all-cause mortality remained significant (HR = 1.80, 95% CI: 1.60–2.03, *P* < 0.001). Second, after excluding participants with a baseline history of CVD or cancer, the relationship between PAA and all-cause mortality remained stable (CVD exclusion group: HR = 1.80, 95% CI: 1.57–2.07, *P* < 0.001; cancer exclusion group: HR = 1.84, 95% CI: 1.58–2.14, *P* < 0.001, Table 3). In Supplementary Table 1, weighted analysis showed that PAA significantly increased the risk of all-cause mortality (HR = 1.90), CVD mortality (HR = 2.03), and cancer mortality (HR = 1.47).

**Table 3:** Sensitivity analyses: HRs (95% CIs) for all-cause mortality among obese adults according to phenotypic age acceleration.

**Characteristics**	**Age acceleration residual**	***P*-value**	**Phenotypic age acceleration**		***P*-value**
			**No**	**Yes**	
**Excluding participants who died with 2 years of follow-up**					
No. deaths/total (%)	1,383/9,038 (15.3)		751/5,354 (14.0)	632/3,684 (17.2)	
Model 1	1.04 (1.04–1.05)	< 0.001	1 (reference)	2.34 (2.10–2.61)	< 0.001
Model 2	1.04 (1.04–1.05)	< 0.001	1 (reference)	2.10 (1.88–2.35)	< 0.001
Model 3	1.04 (1.03–1.04)	< 0.001	1 (reference)	1.80 (1.6–2.03)	< 0.001
**Excluding participant who had a history of CVD at baseline**					
No. deaths/total (%)	999/8,537(11.7)		592/4,972 (11.9)	407/3,565 (11.4)	
Model 1	1.04 (1.04–1.05)	< 0.001	1 (reference)	2.17 (1.90–2.47)	< 0.001
Model 2	1.04 (1.04–1.05)	< 0.001	1 (reference)	1.95 (1.71–2.23)	< 0.001
Model 3	1.04 (1.03–1.04)	< 0.001	1 (reference)	1.80 (1.57–2.07)	< 0.001
**Excluding participant who had a history of Cancer at baseline**					
No. deaths/total (%)	834/7,859 (10.6)		496/4,604 (10.8)	338/3,255 (10.4)	
Model 1	1.04 (1.04–1.05)	< 0.001	1 (reference)	2.25 (1.95–2.59)	< 0.001
Model 2	1.04 (1.03–1.05)	< 0.001	1 (reference)	2.02 (1.75–2.34)	< 0.001
Model 3	1.04 (1.03–1.04)	< 0.001	1 (reference)	1.84 (1.58–2.14)	< 0.001

Model 1 was adjusted for age and gender. Model 2 was additionally adjusted for race, marital status, PIR group, educational level, HEI-2015, physical active, smoking status, and alcohol intake. Model 3 was additionally adjusted for CVD, hypertension, hyperlipidemia, diabetes, and cancer.

HEI-2015, Healthy Eating Index-2015; PIR, poverty income ratio; CVD, cardiovascular disease.

## 4 Discussion

To our knowledge, this study is the first to explore the relationship between PAA and all-cause mortality, CVD mortality, and cancer mortality in the obese population using data from the NHANES. The study found that individuals with PAA exhibited significantly higher risks of all-cause mortality, CVD mortality, and cancer mortality compared to those without PAA. These associations remained significant after adjusting for multiple covariates, indicating that PAA is an independent predictor of mortality in obese individuals. Sensitivity analyses further confirmed the robustness of these findings, providing strong support for the conclusions of this study.

Different BA measurement methods, such as DNA methylation age, telomere length, and Klotho protein levels, have become important tools for assessing BA. Studies on DNA methylation age have shown that it can accurately reflect an individual's biological aging and is closely linked to disease risk (24). Telomere shortening is considered one of the markers of aging and is frequently used to assess an individual's aging state (25). Klotho, an anti-aging protein, has been shown to decrease in concentration with age, and its reduction is linked to various age-related diseases (26). PA, as a novel BA marker, integrates various physiological and biological indicators, offering a more comprehensive prediction of an individual's health status and disease risk (27).

Several previous studies have shown that PAA is an important BA marker in general and disease-specific populations, capable of predicting all-cause and disease-specific mortality. However, most of these studies did not focus on the obese population as a distinct group. A large study of the general adult population revealed a significant positive correlation between PAA and all-cause, cardiovascular, and cancer mortality. The study pointed out that unhealthy lifestyles, such as smoking and lack of physical activity, can accelerate BA and increase mortality risk (28). In disease-specific populations, the role of PAA has also been validated. A study found that PAA significantly predicted long-term mortality in patients with multi-vessel coronary artery disease, serving as a critical prognostic indicator for CVD (10). Similarly, Chen et al. demonstrated that PAA was significantly associated with all-cause and CVD mortality in patients with diabetes, further highlighting its potential value in managing chronic diseases (29). Studies in cancer populations have shown similar results. Wang et al., in a study of cancer survivors, found that PAA significantly increased all-cause mortality, particularly in breast and colorectal cancer survivors. Using a proteomic aging clock (PAC) to assess BA acceleration in cancer survivors, the study showed that faster BA was associated with higher mortality risk (30).

Unlike these studies, the uniqueness of the current study lies in its specific focus on the relationship between PAA and mortality in the obese population. Obesity itself is a major risk factor for chronic diseases such as CVD, diabetes, and cancer. PAA, as a comprehensive BA indicator, can better capture the aging process in obese individuals. While most previous studies have treated obesity as a covariate or subgroup, this study focused on the obese population, revealing the independent predictive value of PAA for mortality in this group. Lundgren et al. examined the relationship between BMI and epigenetic age acceleration, finding that obesity, associated with insulin resistance, accelerated BA (31). This study further demonstrated a strong correlation between PAA and all-cause and cause-specific mortality in obese individuals, expanding the scope of research in this field.

The relationship between PAA and mortality can be explained by several biological mechanisms. First, obesity is considered a trigger for chronic inflammation, and prolonged chronic inflammation can accelerate cellular aging and tissue damage, closely linking it to accelerated aging and increased mortality risk. Zhang et al. pointed out that obesity-induced oxidative stress and inflammatory responses accelerate cellular aging, thereby increasing the incidence of CVD and cancer (32). Second, a study found that the association between obesity and insulin resistance could accelerate BA through metabolic dysregulation, increasing mortality risk in obese individuals (33). Additionally, telomeres, the protective structures at the ends of chromosomes, are known to shorten as a marker of aging. Studies have shown that obesity accelerates telomere shortening, promoting BA. As inflammation and oxidative stress intensify in obese individuals, the rate of telomere shortening may increase further, leading to PAA (34). These mechanisms suggest that obesity accelerates aging through multiple metabolic and biological pathways, which in turn impacts mortality.

This study has several notable strengths. First, it focuses on the obese population, which has important public health implications. Obesity is a global health issue, and clarifying the role of PAA in obese individuals helps better understand the complex relationship between obesity and mortality. Second, this study utilized a large, representative sample with rich covariates, ensuring the robustness and external validity of the findings. Additionally, by adjusting for multiple variables and performing sensitivity analyses, this study confirmed the significant association between PAA and mortality. However, this study also has some limitations. First, the calculation of PAA was based on a one-time measurement and did not capture dynamic changes in an individual's physiological state. Second, despite adjusting for multiple potential confounders, unmeasured confounders may still influence the results. Furthermore, as an observational study, it cannot establish causality between PAA and mortality, and future longitudinal intervention studies are needed to further validate these findings. Finally, grouping multiple ethnicities as “other” may obscure intergroup differences, as varying health risks and socioeconomic factors across ethnic groups could affect the PAA-mortality association. Future research should use more detailed racial classifications to better assess this relationship.

Based on our findings, PAA can serve as a risk assessment tool for obese individuals. Possible interventions include lifestyle modifications, such as increasing physical activity, adopting a healthy diet, and reducing smoking and alcohol intake. Additionally, medical interventions aimed at managing metabolic risk factors and controlling inflammation may help slow biological age acceleration. Clinicians could use PAA as a tool to identify high-risk individuals and develop personalized interventions to reduce mortality risk.

## 5 Conclusion

This study identified PAA as a strong predictor of all-cause, cardiovascular, and cancer mortality in obese individuals, highlighting its importance for the first time in this population. These findings offer new insights for health management and mortality risk assessment in obesity, urging further research into the mechanisms linking PAA to obesity-related diseases.

## Data Availability

The original contributions presented in the study are included in the article/Supplementary material, further inquiries can be directed to the corresponding authors.
