# Supplementary material for: Association between phenotypic age and mortality risk in individuals with obesity: a retrospective cohort study
Source: Front Public Health. 2024 Dec 9;12:1505066. doi: 10.3389/fpubh.2024.1505066 (PMC11663737; doi:10.3389/fpubh.2024.1505066)
Supplement: Supplementary file 1 [file Table_1.docx]

**Supplementary Table 1 Weighted analysis of adjusted hazard ratios of phenotypic age acceleration with risk of all-cause mortality and cause-specific mortality**

| **Characteristics** | **Age acceleration residual** | ***P*-value** | **Phenotypic age acceleration** | | ***P*-value** |
| --- | --- | --- | --- | --- | --- |
|  |  |  | **No** | **Yes** |  |
| **All-cause mortality** |  |  |  |  |  |
| No. deaths/total (%) | 1537/9925(15.5) |  | 793/5496 (14.4) | 744/4429 (16.8) |  |
| Model 1 | 1.05 (1.04-1.05) | <0.001 | 1 (reference) | 2.55 (2.25-2.89) | <0.001 |
| Model 2 | 1.04 (1.03-1.05) | <0.001 | 1 (reference) | 2.20 (1.94-2.49) | <0.001 |
| Model 3 | 1.04 (1.03-1.04) | <0.001 | 1 (reference) | 1.90 (1.63-2.20) | <0.001 |
|  |  |  |  |  |  |
| **CVD** **mortality** |  |  |  |  |  |
| No. deaths/total (%) | 419/9925(4.2) |  | 206/5496 (3.7) | 213/4429 (4.8) |  |
| Model 1 | 1.05 (1.04-1.06) | <0.001 | 1 (reference) | 2.79 (2.18-3.57) | <0.001 |
| Model 2 | 1.05 (1.04-1.06) | <0.001 | 1 (reference) | 2.47 (1.90-3.19) | <0.001 |
| Model 3 | 1.04 (1.03-1.05) | <0.001 | 1 (reference) | 2.03 (1.46-2.81) | <0.001 |
|  |  |  |  |  |  |
| **Cancer** **mortality** |  |  |  |  |  |
| No. deaths/total (%) | 357/9925(3.8) |  | 218/5496 (4.0) | 157/4429 (3.5) |  |
| Model 1 | 1.03 (1.02-1.04) | <0.001 | 1 (reference) | 1.78 (1.41-2.25) | <0.001 |
| Model 2 | 1.03 (1.02-1.03) | <0.001 | 1 (reference) | 1.57 (1.24-1.99) | <0.001 |
| Model 3 | 1.02 (1.01-1.03) | <0.001 | 1 (reference) | 1.47 (1.15-1.88) | <0.001 |

Model 1 was adjusted for age and gender. Model 2 was additionally adjusted for race, marital status, PIR group, educational level, HEI-2015, physical active, smoking status, and alcohol intake. Model 3 was additionally adjusted for CVD, hypertension, hyperlipidemia, diabetes, and cancer.

Abbreviations: HEI-2015, Healthy Eating Index-2015; PIR, poverty income ratio; CVD, cardiovascular disease.

**Supplementary Table 2 Adjusted hazard ratios of phenotypic age acceleration with risk of all-cause mortality and cause-specific mortality among adults with central obesity**

| **Characteristics** | **Age acceleration residual** | ***P*-value** | **Phenotypic age acceleration** | | ***P*-value** |
| --- | --- | --- | --- | --- | --- |
|  |  |  | **No** | **Yes** |  |
| **All-cause mortality** |  |  |  |  |  |
| No. deaths/total (%) | 2766/14886(18.6) |  | 1630/9108 (17.9) | 1136/5778 (19.7) |  |
| Model 1 | 1.04 (1.04-1.04) | <0.001 | 1 (reference) | 2.27 (2.10-2.45) | <0.001 |
| Model 2 | 1.04 (1.04-1.04) | <0.001 | 1 (reference) | 2.03 (1.87-2.2) | <0.001 |
| Model 3 | 1.04 (1.03-1.04) | <0.001 | 1 (reference) | 1.81 (1.66-1.97) | <0.001 |
|  |  |  |  |  |  |
| **CVD** **mortality** |  |  |  |  |  |
| No. deaths/total (%) | 746/14886(5) |  | 435/9108 (4.8) | 311/5778 (5.4) |  |
| Model 1 | 1.04 (1.04-1.05) | <0.001 | 1 (reference) | 2.25 (1.94-2.62) | <0.001 |
| Model 2 | 1.04 (1.03-1.05) | <0.001 | 1 (reference) | 1.98 (1.69-2.32) | <0.001 |
| Model 3 | 1.03 (1.03-1.04) | <0.001 | 1 (reference) | 1.71 (1.45-2.01) | <0.001 |
|  |  |  |  |  |  |
| **Cancer** **mortality** |  |  |  |  |  |
| No. deaths/total (%) | 632/14886(4.2) |  | 403/9108 (4.4) | 229/5778 (4) |  |
| Model 1 | 1.03 (1.02-1.04) | <0.001 | 1 (reference) | 1.76 (1.49-2.08) | <0.001 |
| Model 2 | 1.02 (1.02-1.03) | <0.001 | 1 (reference) | 1.57 (1.32-1.87) | <0.001 |
| Model 3 | 1.02 (1.01-1.03) | <0.001 | 1 (reference) | 1.52 (1.26-1.82) | <0.001 |

The criteria for central obesity: Men, Waist circumference ≥ 102 cm (40 inches); Women, Waist circumference ≥ 88 cm (35 inches).

Model 1 was adjusted for age and gender. Model 2 was additionally adjusted for race, marital status, PIR group, educational level, HEI-2015, physical active, smoking status, and alcohol intake. Model 3 was additionally adjusted for CVD, hypertension, hyperlipidemia, diabetes, and cancer.

Abbreviations: HEI-2015, Healthy Eating Index-2015; PIR, poverty income ratio; CVD, cardiovascular disease.

**Supplementary Table 3 Adjusted hazard ratios of phenotypic age acceleration with risk of all-cause mortality and cause-specific mortality among adults with overweight**

| **Characteristics** | **Age acceleration residual** | ***P*-value** | **Phenotypic age acceleration** | | ***P*-value** |
| --- | --- | --- | --- | --- | --- |
|  |  |  | **No** | **Yes** |  |
| **All-cause mortality** |  |  |  |  |  |
| No. deaths/total (%) | 1713/9250(18.5) |  | 1134/6767 (16.8) | 579/2483 (23.3) |  |
| Model 1 | 1.04 (1.03-1.04) | <0.001 | 1 (reference) | 2.27 (2.10-2.45) | <0.001 |
| Model 2 | 1.03 (1.03-1.04) | <0.001 | 1 (reference) | 2.02 (1.82-2.25) | <0.001 |
| Model 3 | 1.03 (1.03-1.04) | <0.001 | 1 (reference) | 1.83 (1.63-2.05) | <0.001 |
|  |  |  |  |  |  |
| **CVD** **mortality** |  |  |  |  |  |
| No. deaths/total (%) | 456/9250(4.9) |  | 298/6767 (4.4) | 158/2483 (6.4) |  |
| Model 1 | 1.04 (1.03-1.04) | <0.001 | 1 (reference) | 2.34 (1.92-2.85) | <0.001 |
| Model 2 | 1.03 (1.03-1.04) | <0.001 | 1 (reference) | 2.03 (1.65-2.49) | <0.001 |
| Model 3 | 1.03 (1.02-1.04) | <0.001 | 1 (reference) | 1.74 (1.4-2.16) | <0.001 |
|  |  |  |  |  |  |
| **Cancer** **mortality** |  |  |  |  |  |
| No. deaths/total (%) | 398/9250(4.3) |  | 278/6767 (4.1) | 120/2483 (4.8) |  |
| Model 1 | 1.03 (1.02-1.04) | <0.001 | 1 (reference) | 1.86 (1.49-2.32) | <0.001 |
| Model 2 | 1.02 (1.01-1.03) | <0.001 | 1 (reference) | 1.64 (1.31-2.06) | <0.001 |
| Model 3 | 1.02 (1.01-1.03) | <0.001 | 1 (reference) | 1.67 (1.32-2.13) | <0.001 |

The criteria for overweight: BMI between 25.0 and 29.9 kg/m².

Model 1 was adjusted for age and gender. Model 2 was additionally adjusted for race, marital status, PIR group, educational level, HEI-2015, physical active, smoking status, and alcohol intake. Model 3 was additionally adjusted for CVD, hypertension, hyperlipidemia, diabetes, and cancer.

Abbreviations: HEI-2015, Healthy Eating Index-2015; PIR, poverty income ratio; CVD, cardiovascular disease.

**The calculation formula for phenotypic age**

$$Phenotypic age = 141.50 + \frac{Ln[-0.00553 \times Ln(exp(\frac{-1.51714 \times\mathrm{ex}p \left( xb \right)}{0.0076927}))]}{0.09165}$$

where *xb* = − 19.907 − 0.0336 × Albumin (g/L) + 0.0095 × Creatinine (μmol/L) + 0.1953 × Glucose (mmol/L) + 0.0954 × LnCRP (mg/dL) − 0.0120 × Lymphocyte Percent (%) + 0.0268 × Mean Cell Volume (fL) + 0.3306 × Red Cell Distribution Width (%) + 0.00188 × Alkaline Phosphatase (U/L) + 0.0554 × White Blood Cell Count (1000 cells/ μL) + 0.0804 × CA (years)^14^.

**Weighted analysis**

Weighted analysis is a statistical method that assigns different weights to observations in a sample to reflect their importance and representativeness within the overall dataset. This technique enhances the accuracy and efficiency of estimates and allows for a deeper understanding of population characteristics and relationships between variables. By adjusting sample weights, weighted analysis corrects estimation biases, leading to more accurate results. Additionally, it optimizes estimation efficiency by assigning different weights to observations based on their relative importance, ensuring that the sample is representative and leading to more accurate population estimates[1]. Detailed information and methodology regarding NHANES weighting can be found in the NHANES guidelines on their website [2]. Following these guidelines, we incorporated the complex sampling design and mobile examination center sample weights into our study, ensuring that our data sample represents a large and diverse U.S. adult population. The sampling weights were determined as follows: for the 1999–2002 period, weights were calculated as 2/8 × 4-year MEC weight, while for the 2003–2010 and 2015–2018 periods, weights were 1/8 × 2year MEC weight. It is important to note that data for CRP, which are essential for calculating PA, were unavailable for the 2011–2014 period.

**References:**

1. NHANES Survey Methods and Analytic Guidelines. https://wwwn.cdc.gov/nchs/nhanes/analyticguidelines.aspx#estimation-and-weighting-procedures. Accessed 12 Aug 2024

2. NHANES Survey Methods and Analytic Guidelines. https://wwwn.cdc.gov/nchs/nhanes/analyticguidelines.aspx#estimation-and-weighting-procedures. Accessed 12 Aug 2024
